# Neurobiology and the Humanities

**DOI:** 10.1016/j.neuron.2014.09.016

**Published:** 2014-10-01

**Authors:** Semir Zeki

**Affiliations:** 1 University College London, London, WC1E 6BT, UK

## Abstract

Can the arts and humanities contribute significantly to the study of the brain? Similar brain processes are involved in humanistic and scientific inference, and in this essay, I argue that conclusions reached by one are relevant to the other.

## Main Text

Can the arts and humanities contribute significantly to brain studies? Do they frame questions regarding human experience that can be tested experimentally and are these fundamentally different from those posed by neuroscience? Is there any present need or imperative to appropriate questions from them in neurobiological studies, or should that be deferred until more is known about the functions and functioning of the brain? These questions impose themselves forcefully at a time when a significant proportion of human brain studies are addressing questions that are of importance to human experience.

### Common Questions

Science and the humanities have much to separate them but much to unite them too. Artistic and scientific questions are commonly the same, though addressed differently, and hence, the former provide hints and guesses for scientific experimentation. I have written of artists and humanists as being, in a sense, neurobiologists who explore the brain, though with techniques that are unique to them (Zeki, 1993). Paul Cézanne’s preoccupation, and artistic experimentation, with how color modulates form is but a variant of the neurobiological question of how the separate representations of form and color are integrated in the brain to give us a unitary percept of both (Zeki, 1978; Livingstone and Hubel, 1988). The experiments of Picasso and Braque in the early, analytic, phase of cubism—of how a form maintains its identity in spite of wide variations in the context in which it is viewed—resolves itself scientifically into the neurobiological problem of form constancy. The quest of Piet Mondrian for the “constant truths concerning forms” (Mondrian, 1941) is an artistic version of the question of what the neural building blocks of all forms are (often presumed to be the orientation-selective cells of the visual cortex), while kinetic art, which sought to represent motion artistically, reached conclusions that are consistent with conclusions reached later by neurobiology (Zeki and Lamb, 1994).

### All Truths Are Subjective

These are, in a sense, facile rallying points that merely serve to emphasize different approaches to what are, at heart, common questions. More difficult to address are shared questions regarding human experience and what they signify about brain operations and the world in which it has developed. Here the boundary between neurobiological and humanistic questions is faint and separating the two, I believe, does both a disservice even if, at present, the relationship between neuroesthetics and the humanities is asymmetric, in that neuroesthetics has a good deal more to gain from the humanities than the latter from us. Many of the critical questions now addressed experimentally by neuroesthetics have been addressed in philosophical discourse for centuries. Prominent among these is the problem of knowledge, a primordial function of the brain and a central issue in philosophy. Using color vision as an example, Arthur Schopenhauer argued that “a more precise knowledge and firmer conviction of the wholly subjective nature of color contributes to a more profound comprehension of the Kantian doctrine of the likewise subjective, intellectual forms of all knowledge” (Schopenhauer, 1854), since color is a subjective experience that is the result of a transformation of the objective reality of the outside world by rules that govern the operations of the mind (brain). The only knowledge we can therefore have of color is “brain knowledge”. The brain, far from representing colors (or indeed the sensory world) passively and veridically, constructs them through inherited programs (algorithms) (Zeki, 1993). Neurobiology has yet to unravel the details of these operations, but their purpose is to stabilize the colors of surfaces in spite of continual fluctuations in the wavelength-energy composition of the light reflected from them, leading to a constancy of colors. While we can be (subjectively) sure that a leaf is green even when it reflects more long-wave (red) light (as is common at sunset or sunrise), we can never be sure, unless armed with light-measuring devices, of the “objective” reality in terms of the precise wavelength-energy composition of the light reflected from a surface and from its surrounds. Generally speaking, the only truths that we can be certain of are those that we experience, namely subjective truths. This is but one example of a shared general question in neurobiology and the humanities—of how objects and situations maintain their identity in spite of continual changes in the signals reaching the brain from them, summarized for Western philosophy in the Heraclitan doctrine of flux and for Eastern (Buddhist) philosophy in the statement that “nothing is permanent except change.”

### Similar Inferential Processes in Scientific and Humanistic Approaches

The primacy of subjective truths extends from an apparently elementary process such as color to much more complex experiences, such as those of beauty, desire, and love as well as to abstract concepts such as the experience of mathematical beauty. The path to acquiring knowledge—whether grounded in scientific experimentation or through philosophical (Cellucci, 2013) or humanistic speculation—must use similar mental processes. There is no reason to suppose that the brain processes leading to subjective truths—in terms of inference, which is the result of observation and of inductive, deductive, and analogic reasoning—are different for the sciences and the humanities. Indeed, the similarity may extend to metaphoric and metonymic reasoning. The humanistic approach—be it in art or philosophy—is equally grounded in experimentation, of a different, more speculative kind but one that is nevertheless also subject to the logic of the brain. Its results, significantly, lend themselves to scientific experimentation. Hence, in seeking to understand human nature and the human condition, conclusions reached by humanistic debate and discussion are no less or more valid than those reached by scientific experimentation, even if translation from humanistic achievements to scientific experimentation is neither straightforward nor easy. A major difference is that, to attain scientific status as valid for populations instead of individuals, subjective truths require scientific validation, usually through statistical inference. Indeed, given their longevity and the similarity in brain processes leading to inferences in both the sciences and humanities, subjective truths revealed by humanistic discourse can in fact be said to have also been subject to scientific experimentation and statistical validation and hence provide rich material for scientific experimentation. The works of Plato, Sophocles, Kant, Shakespeare, Dostoevsky, and Balzac, among others, have a longevity even surpassing those of scientific works because they reveal subjective truths that are generally applicable to all humans. One is likely to acquire as much experimentally testable knowledge, for example, from reading Kant on aesthetics or Balzac and Zola on creativity than one would from any presently available scientific text.

### The Experience of Beauty

Perhaps nowhere is the interdependence of humanistic enquiry and experimental investigation more intertwined than in the study of one of the most ubiquitous of (subjective) human experiences—that of beauty; it serves as a powerful ground, as well as an example, for uniting the humanistic and neurobiological approaches. Neuroesthetics does not enquire into what beauty is and does not (contrary to common belief) confound it with art. It also acknowledges the importance of culture and learning in shaping aesthetic experience. But its primary concern at present is to understand the neural mechanisms that allow all humans, regardless of race or culture, to experience beauty. Since an aesthetic experience implies having made a judgment, it also aims to unravel the neural systems underlying aesthetic judgments and address the question, first posed by Kant, of whether aesthetic judgments precede or succeed aesthetic experiences. In short, like the art critic Clive Bell, neuroesthetics seeks to understand what, in aesthetic experience, is “common to all and peculiar to none” (Bell, 1914), which is not to deny that, superimposed upon the commonality, there are subjective differences in experiences that science must account for.

It was, after all, a philosopher, Edmund Burke, who defined beauty in significantly neurobiological terms, as being “largely a property of objects acting upon the *human mind* through the *intervention of the senses*” (Burke, 1757, my emphasis). Today, much of the inspiration for the paradigms used to study the neurobiology of aesthetic experience, whether acknowledged or not, comes from philosophical studies.

Though Bell thought of aesthetic experience as a “purely subjective business,” he, like others before and after him, sought for “objective” characteristics that constitute an essential ingredient of beauty. Whether such a characteristic exists has been debated but without a consensus. This is not surprising. Symmetry, for example, is not considered to be characteristic of beauty in all cultures; it does not therefore qualify as a characteristic that is “common to all and peculiar to none.” Characteristics such as proportion or size, though of importance in domains such as architecture, are meaningless when applied to the aesthetics of, for example, color. As well, there is the functional specialization in the brain and in vision, for example, different areas of the (visual) brain are specialized to process different attributes such as color, motion, and form (Zeki, 1978; Zeki et al., 1991). This suggests that, in the visual domain, there may be many different visual characteristics (which I have termed “significant configurations”; Zeki, 2013), belonging to different visual domains, each one capable of activating the relevant visual area in a manner that arouses the “aesthetic emotion” in that domain, as appears to be the case for kinetic stimuli (Zeki and Stutters, 2012). But there is a common characteristic that is independent of learning, culture, and ethnic origin to all that is experienced as beautiful, one that is “common to all and peculiar to none.” It lies in a simple neurobiological fact—that whenever an individual experiences beauty, regardless of whether the source is visual, musical, moral, or mathematical, there is a correlate in the form of activity in a part of the emotional brain, namely field A1 of medial orbitofrontal cortex (A1 mOFC) (Ishizu and Zeki, 2011). Interestingly, this area is also active when subjects have pleasant or rewarding experiences—both of which have been strongly linked to beauty in the philosophy of aesthetics (Gordon, 1997), providing a good area for future experimentation designed to reveal the relationship, in neural terms, between these subjective experiences. This raises the question of what role the sensory areas of the brain play in translating significant visual configurations into an aesthetic emotion, a neurobiological problem of importance that extends well beyond neuroesthetics. Whether stimuli such as faces, for example, are perceived as ugly or beautiful, they activate common areas critical for the perception of faces (Kanwisher et al., 1997; Haxby et al., 2000). But faces that are perceived as beautiful correlate as well with activity in mOFC (O’Doherty et al., 2003), while those experienced as fearful correlate with activity in amygdala (Morris et al., 1996). Some feature of these stimuli must activate the common areas differentially, leading to different outputs from them. Neurobiologically, the question resolves itself into the broader one of the pattern of activity within a common area that dictates the selective output from it to one destination or another.

### The Larger Significance of Beauty

If all truths, whether sensory, aesthetic, or derived from higher cognitive and intellectual sources such as mathematics are subjective, it becomes interesting to ask whether the (subjective) experience of beauty in general is a pointer to universal truths about ourselves and the Universe in which we have evolved, just as sensory experiences such as those of color are pointers to truths about ourselves and the ever fluctuating world in which we have evolved. The experience of color, derived from a sensory source, reveals a truth about how our brain obtains knowledge by stabilizing the continually changing world in which it has evolved sensorially. That the experience of mathematical beauty, just like the experience of musical and visual beauty, correlates with activity in field A1 of mOFC not only shows the abstract nature of beauty but also raises the question of whether beauty, regardless of its source, is also a pointer to deeper truths, a sort of yardstick for determining the truthfulness of what that experience reveals. Put simply, to what extent is the structured order, or the ordered structure, of the Universe in which we have evolved reflected in the organization of our brains and to what extent is the experience of beauty a pointer to that structure? Beauty, which lies at the heart of these questions, is a topic that has traditionally been more speculated on in humanistic debate, though one that is becoming of increasing interest to science and especially neurobiology. A neurobiological quest for what enables us to experience beauty and what that experience signifies is vastly impoverished without significant reliance on speculations in the humanities.

### The Many Uses of Beauty

In *The Descent of Man*, Charles Darwin made sexual selection the centerpiece of his views on beauty and there seems little doubt that, for example, plumage on male birds, often perceived as beautiful by humans, reveals a (subjective) truth in the females about desirable male characteristics in that species, making the bearer a suitable mate for reproductive selection. But, as Rothenberg (2011) has emphasized, this raises the question of why a particular combination of colors is chosen, and why particular structural patterns are used by, for example, bowbirds to create their bowers to attract females. Basing beauty on sexual selection alone also leaves out of account other examples of beauty such as camouflage, which have functions the opposite of attracting sexual attention (Rothenberg, 2011). Hence, an enquiry into why particular patterns or colors are chosen to act as sexual attractors also constitutes an enquiry into whether what is experienced as beautiful is related as well to what coincides with patterns in our brain, which has evolved to construct a picture of the external world.

That fundamental laws governing the structure of our Universe can be expressed in mathematical formulations that arouse the “aesthetic emotion” has long been emphasized by mathematicians, who in general place a high premium on beauty. Plato and the Platonic tradition suppose that mathematical formulations are experienced as beautiful because they give insights into the fundamental structure of the Universe and hence its beauty. Kant went beyond and supposed that such formulations arouse the aesthetic emotion because of the feeling that “they make sense” (Breitenbach, 2013). What “makes sense” is of course what corresponds to the workings and above all the logic of the brain. Hence the aesthetic emotion, even in the “queen of sciences,” may be a pointer as much toward truths about both the Universe as about the workings of the brain. It leads one to enquire, for example, whether humans would have developed string theory, for which there is little if any experimental evidence, if we did not possess the kind of brain organization that we have. It is a fascinating question.

In summary, once we acknowledge that all knowledge is mediated through the operations of the brain and its cognitive apparatus, and is therefore subjective, and that similar brain processes are involved in humanistic and scientific inference, we are led ineluctably to the view that conclusions reached by one are relevant to those reached by the other and that the humanities provide a rich source of hints about the operations of the brain, which neurobiology and more particularly neuroesthetics should be ready to exploit and is doing so.
